# Will Adoption of the 2010 WHO ART Guidelines for HIV-Infected TB Patients Increase the Demand for ART Services in India?

**DOI:** 10.1371/journal.pone.0024297

**Published:** 2011-09-08

**Authors:** Ajay M. V. Kumar, Devesh Gupta, B. B. Rewari, Damodar Bachani, Suresh Mohammed, Vartika Sharma, Kumaraswamy Lal, H. R. Raveendra Reddy, Balaji Naik, Rita Prasad, Mohammed Yaqoob, K. G. Deepak, Suresh Shastri, Srinath Satyanarayana, Anthony David Harries, Lakhbir Singh Chauhan, Puneet Dewan

**Affiliations:** 1 Central TB Division, Directorate General of Health Services, Ministry of Health and Family Welfare, Government of India, New Delhi, India; 2 Office of the World Health Organization (WHO) Representative to India, New Delhi, India; 3 National AIDS Control Organisation, Ministry of Health and Family Welfare, Government of India, New Delhi, India; 4 State TB Cell, Directorate General of Health Services, Ministry of Health and Family Welfare, Government of Karnataka, Bangalore, India; 5 Karnataka State AIDS Prevention and Control Society, Ministry of Health and Family Welfare, Government of Karnataka, Bangalore, India; 6 International Union against Tuberculosis and Lung Diseases (The Union), South East Asia Regional Office, New Delhi, India; 7 International Union Against Tuberculosis and Lung Diseases (The Union), Paris, France; 8 London School of Hygiene and Tropical Medicine, London, United Kingdom; Institute of Infectious Diseases and Molecular Medicine, South Africa

## Abstract

**Background:**

In 2010, WHO expanded previously-recommended indications for anti-retroviral treatment to include all HIV-infected TB patients irrespective of CD4 count. India, however, still limits ART to those TB patients with CD4 counts <350/mm^3^ or with extrapulmonary TB manifestations. We sought to evaluate the additional number of patients that would be initiated on ART if India adopted the current 2010 WHO ART guidelines for HIV-infected TB patients.

**Methods:**

We evaluated all TB patients recorded in treatment registers of the Revised National TB Control Programme in June 2010 in the high-HIV prevalence state of Karnataka, and cross-matched HIV-infected TB patients with ART programme records.

**Results:**

Of 6182 TB patients registered, HIV status was ascertained for 5761(93%) and 710(12%) were HIV-infected. 146(21%) HIV-infected TB patients were on ART prior to TB diagnosis. Of the remaining 564, 497(88%) were assessed for ART eligibility; of these, 436(88%) were eligible for ART according to 2006 WHO ART guidelines. Altogether, 487(69%) HIV-infected TB patients received ART during TB treatment. About 80% started ART within 8 weeks of TB treatment and 95% received an efavirenz based regimen.

**Conclusion:**

In Karnataka, India, about nine out of ten HIV-infected TB patients were eligible for ART according to 2006 WHO ART guidelines. The efficiency of HIV case finding, ART evaluation, and ART initiation was relatively high, with 78% of eligible HIV-infected patients actually initiated on ART, and 80% within 8 weeks of diagnosis. ART could be extended to all HIV-infected TB patients irrespective of CD4 count with relatively little additional burden on the national ART programme.

## Introduction

HIV-infected TB patients experience a high case-fatality rate during anti-tuberculosis (TB) treatment[1]. Antiretroviral therapy (ART) reduces this risk of death, provided ART is started early enough during anti-TB treatment[2]. The efficient and timely initiation of ART in HIV-infected TB patients is crucial to reduce mortality among HIV-infected TB patients.

In India, the National AIDS Control Programme (NACP) ART guidelines are still in line with 2006 World Health Organization (WHO) Guidelines for ART[3], [4]. As per 2006 guidelines, all HIV-infected persons with extra pulmonary (EPTB) or disseminated TB and HIV-infected pulmonary tuberculosis (PTB) patients with a CD4-lymphocyte count ≤350/mm^3^ are considered eligible for ART. In contrast, the 2010 WHO ART Guidelines recommend that ART be initiated in all HIV-infected TB patients (PTB and EPTB), irrespective of CD4 count, as soon as possible during the initial phase of anti-TB treatment[5].

India proposes to change over to the new 2010 WHO ART Guidelines, but there is concern that giving ART to all HIV-infected TB patients may have major resource implications for the national ART programme. There is little information available about what proportion of HIV-infected TB patients are eligible for ART according to the current NACP criteria and how this might change if the new 2010 WHO ART Guidelines were adopted and implemented in India. There was some information on ART eligibility from a previous study, but this was not representative as most of the HIV-infected TB patients could not be evaluated for ART eligibility because of poor information on CD4 counts [6]. Better knowledge of ART eligibility and the efficiency with which patients are initiated on ART is critical for policy makers to plan for increases in demand for drugs and services.

We conducted a cross-sectional survey in the state of Karnataka, one of the highest HIV prevalent states in India, to evaluate the additional number of patients that would be initiated on ART if India adopted current WHO ART guidelines for HIV-infected TB patients. The specific objectives were to assess:- i) number (proportion) of TB patients ascertained for HIV status with their results, ii) number (proportion) of HIV-infected TB patients eligible for ART and started on ART during anti-TB treatment and iii) when ART was started and the type of regimen used.

## Methods

### Design

This was a cross sectional study involving review of routinely collected data recorded in TB and HIV programme records.

### Setting

Karnataka, a south Indian state with a population of 61 million, has an estimated 0.25 million people living with HIV in 2009 and accounts for about 10% of country's HIV burden[7]. Hence, the state has been classified as ‘high priority’ for HIV interventions by the Indian National AIDS Control Organization (NACO) on the basis of consistently high HIV seroprevalence rates of >1% during sentinel surveillance at antenatal clinics[8], [9]. In the state, tuberculosis control programme services are available through a decentralized network of primary health care facilities which provide general health services including diagnosis and treatment for TB. All TB patients are treated with standardized fully intermittent thrice-weekly short-course regimens (6-9 months) administered under direct observation and are registered at one of the 125 sub-district level TB programme management units according to Indian programme guidelines[10].

There is a national policy of Provider-Initiated HIV Testing and Counseling (PITC) of all TB patients, and HIV-infected TB patients are provided cotrimoxazole preventive therapy (CPT) and referred to ART centres for assessment of ART eligibility and initiation on ART if found to be eligible[11]. TB patients are referred for free HIV counseling and testing to one of the 1000 integrated counseling and testing centre (ICTC) throughout the state, which are usually co-located with sputum microscopy services. Free ART is provided through a network of 40 ART centres (with at-least one ART centre in every district), where HIV-infected patients (including TB patients) are screened for ART eligibility and offered treatment and care. These service delivery sites under NACP follow the national guidelines for counseling, testing, care and treatment of HIV-infected patients[12]. In the year 2010, of 68,655 TB patients registered in Karnataka, 82% were tested for HIV and 8,485 HIV-infected TB patients were identified[13].

### Study population

From a cohort of TB patients (except transfer-in cases) registered in Karnataka state, India, between 1^st^ and 30^th^ June 2010, all those identified as HIV-infected were consecutively included in the study.

### Data collection and validation

Data on the number of TB patients registered, number with known HIV status and number HIV-infected were extracted from the TB registers. TB-HIV data were collected into a pre-tested structured data abstraction form by trained consultants between November and December 2010; data collected included age, sex, type and category of TB, CD4 count, ART initiation, timing of ART initiation and ART regimen. These data were collected from the existing programme records of Revised National TB Control Programme (TB registers and TB treatment cards) and NACP (TB/HIV register, pre-ART registers, ART registers and the electronic patient database maintained at the ART centres). If HIV-infected TB patients were receiving ART from another district, the records of the respective ART centres were reviewed. If there were discrepancies between data in the different records, these were resolved by interviewing the medical officers of the respective health institution. Except where mentioned, standard definitions according to the national programme guidelines were used.

### Data entry and analysis

Data were double-entered into an EpiData database by two data entry operators independently[14]. Databases were compared and discrepancies resolved through referral to the original questionnaire. Proportions of TB patients with known HIV status and proportions of HIV-infected TB patients who were eligible for ART, initiated on ART with timing of ART initiation were calculated.

### Ethics approval

Ethics approval was obtained by the Ethics Advisory Group of the International Union against TB and lung disease (The Union). Approvals of Central TB Division, Ministry of Health and Family Welfare and National AIDS Control organization were also obtained for conducting this evaluation.

## Results

Of 6,182 TB patients registered in Karnataka in June 2010, HIV status was known for 5,761 (93%), and 710 (12%) were recorded as HIV-infected. The demographic, clinical and immunological characteristics of HIV-infected TB patients are shown in **Table 1**. Two thirds of the patients were men, with the majority being in the age group 25–54 years. Most of the patients had new TB and about three quarters of patients were classified as pulmonary tuberculosis with almost equal division between sputum-smear positive and sputum smear negative. CD4-counts were recorded for 621 (87%) patients, of whom 512 (82%) had a CD4 count less than or equal to 350 cells/mm^3^.

**Table 1:** Characteristics of HIV-infected TB patients registered in Karnataka state, India, in June 2010.

Category	Sub-category	Number (%)
All patients		710 (100)*
Sex	Male	465 (66)
	Female	245 (34)
Age group	0–14 years	33 (5)
	15–24 years	23 (3)
	25–35 years	214 (30)
	35–44 years	283 (40)
	45–54 years	114 (16)
	55–64 years	38 (5)
	≥65 years	5 (1)
Type of TB**	Pulmonary TB	516 (73)
	Extra pulmonary TB	194 (27)
Smear status of Pulmonary TB (N = 516)	Smear Positive	285 (55)
	Smear Negative	216 (42)
	Smear Unknown	15 (3)
Site of Extra pulmonary TB (N = 194)	Lymph Node	62 (32)
	Pleura	39 (20)
	Meninges/CNS	33 (17)
	Abdomen	30 (16)
	Other	7 (3)
	Not recorded	23 (12)
TB registration type	New	593 (83)
	Relapse	30 (4)
	Treatment after Default	19 (3)
	Treatment after Failure	5 (1)
	Other	63 (9)
CD4 cell count	<50/mm^3^	72 (10)
	50–200/mm^3^	291 (41)
	201–350/mm^3^	149 (21)
	>350/mm^3^	109 (15)
	Not available	89 (13)

*Percentages may not always add up to 100 due to rounding errors.

**There were 10 patients who had both pulmonary and extrapulmonary TB; they have been classified under extrapulmonary TB as they belong to HIV stage 4 and are ART eligible.

TB-Tuberculosis; HIV-Human immunodeficiency virus; CNS-Central Nervous System.

**Figure 1:**
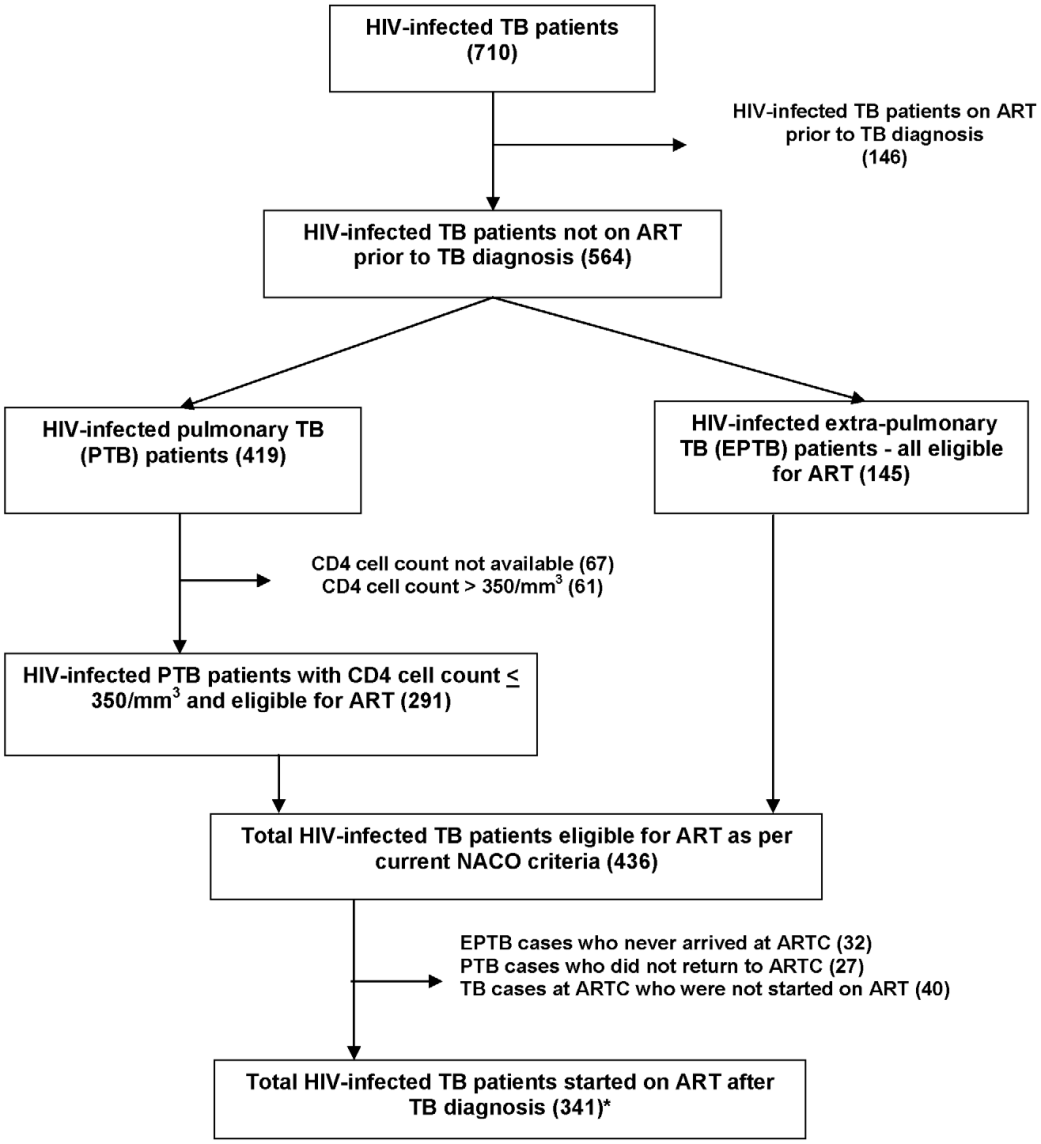
Eligibility for and initiation of antiretroviral treatment in HIV-infected TB patients registered in Karnataka state, India, in June 2010. *4 PTB patients were started on ART even though not eligible as per guidelines TB-Tuberculosis; HIV-Human immunodeficiency virus; PTB-pulmonary tuberculosis; EPTB-extrapulmonary tuberculosis; NACO-National AIDS Control Organization; ART-antiretroviral treatment; ARTC-ART centre.

Assessment for ART eligibility and initiation of ART is shown in **Figure 1**. Of all 710 HIV-infected TB patients identified, 146 (21%) were already on ART prior to TB diagnosis. Of the 564 patients who were not on ART at the time of TB diagnosis, 145 (25%) had extra-pulmonary TB and were immediately ART eligible. Of the 419 pulmonary TB patients not already on ART, 352 (84%) had available CD4 information, among whom 291 (83%) had a CD4 count <350 cells/mm^3^ and were ART eligible. Altogether, 497/564 (88%) could be assessed for ART eligibility and among them, 436 (88%) were eligible for ART.

Overall, 487(67%) HIV-infected TB patients received ART during TB treatment, with ART started either before or after the diagnosis of TB.

For those patients who received ART either before or after TB diagnosis, the time of initiation of ART and the type of regimen are shown in **Table 2**. Of the 341 patients who started ART after TB diagnosis, 272 (80%) started within eight weeks of commencing anti-TB treatment. Almost 95% of patients received an efavirenz-based ART regimen.

**Table 2:** Timing of ART initiation and ART regimen in HIV-infected TB patients registered in Karnataka state, India, in June 2010.

Category	Sub category	Number (%)
All patients on ART		487 (100)
Timing of ART initiation	Prior to TB diagnosis	146 (30)
	<2 weeks of TB treatment	67 (14)
	2–8 weeks of TB treatment	205 (42)
	>8 weeks of TB treatment	69 (14)
ART regimen	Zidovudine-Lamivudine-Efavirenz	224 (46)
	Stavudine-Lamivudine-Efavirenz	235 (48)
	Zidovudine-Lamivudine-Nevirapine	9 (2)
	Stavudine-Lamivudine-Nevirapine	13 (3)
	Other/Not recorded	6 (1)

TB-Tuberculosis; HIV-Human immunodeficiency virus; ART-antiretroviral treatment.

## Discussion

This study in Karnataka state showed the majority of TB patients in a high HIV prevalence state in India were tested for HIV, and of those found to be HIV-positive nearly 90% were eligible for ART according to current NACP and 2006 WHO ART guidelines. As a public health approach to ART, this strongly justifies the adoption of the WHO 2010 ART Guidelines, recommending that all HIV-infected TB patients are started on ART regardless of CD4 count. As <10% of HIV-infected TB patients had CD4 counts > 350 cells/mm^3^, the adoption of the WHO ART Guideline recommendation for HIV-infected TB patients is not likely to place a large additional burden on the national ART programme.

We also determined sub-optimal programme efficiency in the initiation of ART for patients eligible under current guidelines; 23% of HIV-infected TB patients should have got ART, but did not. To illustrate this point, we can apply these findings to the state of Karnataka. In the state of Karnataka in 2010, of 68,665 tuberculosis patients registered for treatment, 56,622 knew their HIV status and 8,485 were identified as HIV-infected[13]. Assuming existing operational efficiency, 67% (5,685) would be receiving ART and another 23% (1,951) would be eligible for ART as per current ART eligibility criteria, but not yet on ART due to operational challenges. Increasing the programme efficiency of ART initiation could place 1,951 additional people on ART. About 10% (849) would be eligible for ART if new guidelines are implemented. Given that there were about 55,102 people alive and on ART in the state of Karnataka at the end of 2010, the annual addition of 849 (1.5%) people to the ART programme from ‘changed guidelines’, and 1,951 (3.5%) people from ‘improved programme efficiency’ would be modest. Improvements in programme efficiency of ART initiation for HIV-infected TB patients offers another important opportunity to improve ART coverage in HIV-infected TB patients, and in the larger context of ART provision would pose little additional burden on existing HIV treatment services.

This was the first state-wide study in India assessing the management of HIV diagnosis and care in TB patients under programmatic settings. The only other study to examine this issue in India found similar results in 2 districts of South India, in which about 83% of HIV-infected TB patients were eligible for ART; but this finding suffered from very low completion of CD4 evaluation in that cohort[6]. Similar findings have been reported from sub-Saharan Africa, where 90% of HIV-infected TB patients had CD4 counts below 350 cells/mm^3^ [15]. Our study covered the entire state of Karnataka and had very high rates of HIV case finding among TB patients and CD4 testing. Hence, these results are likely to be broadly generalizable. Given the similarity of results from various settings, we also believe that the results may be extrapolated to other high HIV prevalent states in the country, though more research is required to confirm this. The high rates of HIV testing among TB patients in this cohort could be attributed to the widespread availability of HIV testing services co-located with the TB microscopy centres in the state. Since TB patients can be a high-yield source of HIV case finding, this calls for scale-up of co-located HIV testing and TB testing services across the country.

This study also emphasizes that in resource-poor settings, the majority of HIV-infected TB patients who are not already on ART present to health services late and with low CD4 counts. The reasons for late presentation are many and include late diagnosis of HIV and the low CD4 threshold currently used for initiating ART among all HIV-infected persons. Hence, the national programmes should explore other opportunities of improved and earlier diagnosis of HIV-infected TB patients like PITC of TB suspects and strengthening intensified TB case finding activities at HIV care settings by the use of WHO-endorsed, new, rapid molecular technologies to diagnose TB[16]. The 2010 WHO ART guidelines which recommends initiation of ART for all PLHIV with CD4 count of ≤350/mm^3^ is a welcome change in this regard which may not only prevent TB cases from occurring but may also ensure that those who develop TB would have higher CD4 counts and consequently the likelihood of better clinical outcomes. NACP and RNTCP are considering all the above options in their next joint national strategic plan (2012-17) to achieve improved and earlier diagnosis of HIV-infected TB patients[17].

This study also revealed operational weaknesses in ART services. There were various reasons for poor ART uptake that included failure to reach the ART centre, not getting CD4 counts done, having a CD4 count > 350 cells/uL in the case of pulmonary tuberculosis and not returning to the ART centre after starting TB treatment. Some of these patients may have died or been too sick to reach the ART centre. Removal of the CD4 count hurdle as part of ART eligibility criteria may make it easier to start ART. More than one in five patients defined as ART eligible still failed to receive ART during TB treatment. As per NACP guidelines, patients were required to show proof of address and needed to make 2–3 visits for baseline investigations and adherence counseling before initiation of ART. Though this was important to ensure long term adherence to treatment, it might have contributed to losing track of even those who were eligible for ART. An assessment of reasons for the lack of ART initiation for eligible patients was beyond the scope of the current study and this needs further investigation. On a positive note, the majority of patients who started ART did so within 8 weeks of commencing TB treatment in accordance with the NACP and WHO recommendations, with efavirenz-based ART regimens being those most commonly used as per current NACP guidelines. Early ART initiation for HIV-infected TB patients has been convincingly shown to save lives, and the timeliness of ART initiation should be monitored to ensure continued effective implementation.

This study had some limitations mainly related to our reliance on data collected routinely by the national TB and HIV programmes, where high accuracy of recording is difficult to ensure. Although the 2010 WHO ART guidelines are justified as a public health approach, it is important to study the TB treatment outcomes among the subgroup of HIV-infected TB patients with CD4 count of >350/mm^3^ and assess if they actually benefit by early initiation of ART. A previous study from Cote d'Ivoire indicated that although mortality was less among HIV-infected TB patients with CD4 count of >200/mm^3^ when compared with those whose CD4 count was <200/mm^3^, it still remained many times higher than in HIV-negative TB patients[18]. Information on mortality in HIV-infected TB patients whose CD4 counts of >350/mm^3^ is limited and this needs further study. Further, we did not extend our research to do a costing exercise, but this can and should be a subject of further analysis.

In conclusion, this study indicates that about 90% of HIV-infected TB patients are eligible for ART as per 2006 WHO ART guidelines. Hence as a public health approach, this strongly justifies the adoption of 2010 WHO ART guidelines which recommends that all HIV-infected TB patients should be initiated on ART irrespective of CD4 count; this policy change should have few additional resource implications for the national ART programme. However, systematic measures need to be taken by the national TB and HIV programmes to improve access of HIV-infected TB patients to ART and bridge the gap in ART uptake.
